# Targeting senescent cells in aging and COVID-19: from cellular mechanisms to therapeutic opportunities

**DOI:** 10.1186/s13619-024-00201-1

**Published:** 2024-10-02

**Authors:** Yuan Yu, Kaixuan Lin, Haoyu Wu, Mingli Hu, Xuejie Yang, Jie Wang, Johannes Grillari, Jiekai Chen

**Affiliations:** 1 Center for Cell Lineage and Atlas, Guangdong Provincial Key Laboratory of Stem Cell and Regenerative Medicine, Guangdong-Hong Kong Joint Laboratory for Stem Cell and Regenerative Medicine, Guangzhou Institutes of Biomedicine and Health, Chinese Academy of Sciences, Guangzhou, 510530 China; 2 University of Chinese Academy of Sciences, Beijing, 100049 China; 3 Science and Technology Innovation Center, Guangzhou University of Chinese Medicine, Guangzhou, 510405 China; 4 Austrian Cluster for Tissue Regeneration, Vienna, Austria; 5 Institute of Molecular Biotechnology, BOKU University, Vienna, Austria; 6 Ludwig Boltzmann Institute for Traumatology, The Research Center in Cooperation With AUVA, 1200 Vienna, Austria

**Keywords:** COVID-19, Aging, Cellular Senescence, Anti-aging Therapies

## Abstract

The COVID-19 pandemic has caused a global health crisis and significant social economic burden. While most individuals experience mild or non-specific symptoms, elderly individuals are at a higher risk of developing severe symptoms and life-threatening complications. Exploring the key factors associated with clinical severity highlights that key characteristics of aging, such as cellular senescence, immune dysregulation, metabolic alterations, and impaired regenerative potential, contribute to disruption of tissue homeostasis of the lung and worse clinical outcome. Senolytic and senomorphic drugs, which are anti-aging treatments designed to eliminate senescent cells or decrease the associated phenotypes, have shown promise in alleviating age-related dysfunctions and offer a novel approach to treating diseases that share certain aspects of underlying mechanisms with aging, including COVID-19. This review summarizes the current understanding of aging in COVID-19 progression, and highlights recent findings on anti-aging drugs that could be repurposed for COVID-19 treatment to complement existing therapies.

## Background

Coronavirus disease 2019 (COVID-19) refers to a respiratory infectious disease caused by a novel coronavirus named severe acute respiratory syndrome coronaviruses 2 (SARS-CoV-2). Coronavirus has caused several public health crises in recent years, including severe acute respiratory syndrome (SARS), Middle East respiratory syndrome (MERS) and COVID-19, but our knowledge on how to effectively manage them remains limited (Cui et al. 2019). According to World Health Organization (WHO) statistics, COVID-19 has caused over 774 million confirmed cases and more than 7 million deaths globally (World Health Organization 2024). Although most people are asymptomatic or experienced mild symptoms including headache, fever, cough, fatigue, and loss of smell, some may develop life-threatening complications such as acute respiratory distress syndrome and multi-organ damages (Berlin et al. 2020). The severity of COVID-19 infection depends on a variety of factors, with elderly individuals and those with pre-existing medical conditions, many of which are age-associated, such as diabetes, cardiovascular disease, and chronic lung or kidney diseases, being at greater risk of severe illness (Starke et al. 2021). More than 80% of global COVID-19–related deaths between 2020 and 2021 occurred among people aged 60 years or older (Harris 2023). Similar to COVID-19, aging can cause numerous changes at the molecular, cellular and systemic levels, which are linked to age-related illnesses and increased susceptibility to infectious diseases (López-Otín et al. 2023). Chronic inflammation and immune dysregulation are key features of aging, where accumulation of senescent cells contributes to an excessive release of cytokines known as the senescence-associated secretory phenotype (SASP) (Bajaj et al. 2021). Overproduction of pro-inflammatory cytokines is a major cause of severe COVID-19 cases (Tay et al. 2020). Such a hyperactive inflammatory state not only exacerbates the severity of infections like COVID-19 but also causes complications in other age-related diseases (López-Otín et al. 2023). Therefore, a better understanding of the pathophysiology of aging will help to understand age-related diseases and guide targeted management strategies for other deadly infectious diseases like COVID-19.

## Overview of COVID-19 pathogenesis

SARS-CoV-2 is a novel strain of coronavirus responsible for the COVID-19 pandemic and is the seventh known coronavirus contagious to humans. SARS-CoV-2 is a positive-sense single-stranded RNA virus belonging to *Betacoronavirus* genus*,* which also includes pathogens such as SARS-CoV and MERS-CoV, known for causing previous outbreaks of respiratory diseases (Cui et al. 2019). The spike protein on the SARS-CoV-2 envelop binds to angiotensin-converting enzyme 2 (ACE2), the receptor protein on host cells, and fuses with the host cell membrane via the cell surface non-endosomal and/or endosomal pathways to enter host cells (V’kovski et al. 2021). Once the viral RNA genome is released into the host cell cytoplasm, it initiates viral replication to produce more copies of viral particles (V’kovski et al. 2021).

SARS-CoV-2 infection triggers a rapid inflammatory response within the host. Pattern recognition receptors such as RIG-I, MDA-5, and TLR sense the viral components and trigger the release of cytokines, including IL-1β, IL-6, IL-10, IL-17, TNFα, and type I IFN (Maison et al. 2023). The pro-inflammatory cytokines help stimulate, recruit, and proliferate immune cells and initiate adaptive immune response to fight the virus (Maison et al. 2023). However, the host inflammatory response acts a double-edged sword. The released cytokines create a positive feedback loop that further recruit immune cells, potentially leading to a condition called “cytokine storm”, characterized by excessive cytokine release and associated with severe outcome of COVID-19 (Montazersaheb et al. 2022). The excessive cytokine release can cause life-threatening complications including acute respiratory distress syndrome (ARDS) and multi-organ damages such as cardiac, renal, hepatic, and hemorrhagic damages (Zhang et al. 2020).

Elderly individuals and those with underlying health conditions are more endangered to suffer severe outcome from COVID-19. Understanding the characteristics of aging and their impact on disease susceptibility is crucial for addressing disease vulnerabilities and developing targeted therapies. The following section delves into the interplay between aging and the pathophysiology of COVID-19.

## Characteristics of aging

Aging describes the progressive, general decline in physiological functions over time, a complex phenomenon that involves multifaceted changes at the molecular, cellular, and systemic levels (López-Otín et al. 2023) In a widely recognized review, twelve hallmarks of aging have been described: genomic instability, telomere attrition, epigenetic alterations, loss of proteostasis, disabled macroautophagy, deregulated nutrient-sensing, mitochondrial dysfunction, cellular senescence, stem cell exhaustion, altered intercellular communication, chronic inflammation, and dysbiosis (López-Otín et al. 2023). Recent research continues to explore how these hallmarks contribute to our understanding of the aging process and age-related diseases (Gasek et al. 2021).

Cellular senescence is a major hallmark of aging defined by an irreversible exit from cell cycle triggered by various endogenous or exogenous stressors, including telomere shortening, accumulation of DNA damage, oxidative stress, oncogene activation, and mitochondrial dysfunction (Mylonas and O’Loghlen 2022). Guidelines on how to identify senescence have recently put forward as minimal information on senescence experimentation in vivo (MICSE) (Ogrodnik et al. 2024). Senescent cells resist apoptosis and instead upregulate pro-survival pathways and display a metabolic active hypersecretory phenotype termed the SASP, producing factors including cytokines, interleukins, growth factors, proteases, lipids, and other bioactive substances (Wang et al. 2024), as well as extracellular vesicles and their miRNA cargo (Weilner et al. 2016; Terlecki-Zaniewicz et al. 2018). Although the molecular mechanisms of SASP activation are not fully understood yet, NF‐κB and C/EBP‐β have been identified to be key positive regulators, as well as mTOR and autophagy pathways. Recognition of senescence-associated damage-associated molecular patterns (DAMPs) through Toll-like receptor 2 (TLR2) also regulates the expression of SASP and reinforces cell cycle arrest (Hari et al. 2019). SASP signaling is further amplified by a positive-feedback loop via the activation of the IL-1 signaling pathway the extracellular space stimulate other SASP interleukins, such as IL-6, IL-8, TGF-beta, leading to paracrine pro-senescence and pro-inflammatory response (Orjalo et al. 2009; Ortiz-Montero et al. 2017).

Metabolic changes are associated with aging and many age-associated diseases such as atherosclerosis, type 2 diabetes, non-alcoholic fatty liver disease (Hotamisligil and Erbay 2008). For example, hyperactive mammalian target of rapamycin (mTOR) pathway triggers a series of signals that lead to the unfolded protein response, activation of JUN N-terminal kinase and increase of cytokine secretion (Hotamisligil and Erbay 2008). Small molecular compounds targeting nutrient sensing pathways such as sirtuins, AMP kinase, mTOR and insulin–IGF1 have become major targets for senotherapies. Additionally, lifestyle intervention on metabolism, such as physical activity and calorie restriction, has been shown to contribute to healthier lifespan (Weichhart 2018).

## Role of aging in lung regeneration during COVID-19

The aging lungs undergo several structural changes including progressive loss of alveolar surface area, dilation of air spaces, reduced mucociliary clearance, and decreased elasticity, which contribute to a decline in lung function, and exacerbate the impact of COVID-19 to the elderly (Sharma and Goodwin 2006). Understanding the mechanisms underlying age-related lung regeneration and repair is crucial for developing effective treatments (i.e., senomorphics) to address long-term consequences of COVID-19 on pulmonary function, particularly in the aging population.

The decline of lung-resident stem cells with age compromises the lungs’ ability to repair and regenerative capacity, causing vulnerability to pulmonary diseases (Kotton and Morrisey 2014). Basal cells, which are multipotent progenitors, have the ability to self-renew and differentiate into various cell types, including club cells, goblet cells, neuroendocrine cells, and ciliated cells, decrease in the regenerative potential in the elderly (Schneider et al. 2021; Hong et al. 2004). For instance, Aros et al. demonstrated that glandular-like epithelial invaginations (GLEIs), derived from airway basal stem cells, emerge exclusively in the intercartilaginous zone (ICZ) of aged mice. As airway basal stem cell-derived Wnts facilitate differentiation into ciliated cells in young individuals, the reduced regenerative potential in elderly individuals contributes to epithelial dysfunction in COVID-19 patients (Aros et al. 2020). Additionally, He et al. identified deficiencies in the structural components of cilia and dysregulation of ATP production in the cilia of COVID-19 patients. This results in ciliary dysfunction and impaired mucus clearance (He et al. 2020). Consequently, mucus accumulation can spread infection and enhance inflammation, leading to acute lung injury and acute respiratory distress syndrome (ARDS). The failure to repair airway ciliary dysfunction drives mucus accumulation, as observed in diseases such as primary ciliary dyskinesia, cystic fibrosis, and chronic obstructive pulmonary disease (COPD) (Bustamante-Marin and Ostrowski 2017; Tilley et al. 2015). Moreover, detection of senescent cells in the lungs increased with age, changing the interaction between lung-resident cells, neighboring cells, and the extracellular matrix (ECM) component, leading to increased cytokine production, tissue homeostasis disruption and elastic property changes of the lung (Calhoun et al. 2016). Therefore, aging diminishes the regenerative and repair capacity of human lungs, as well as accelerates senescence-associated secretory phenotype, making it a significant risk factor for severe outcome in COVID-19.

## Long-term consequence of COVID-19 and aging

Long-term consequences of COVID-19, often referred to as "long COVID" or post-acute sequelae of SARS-CoV-2 infection, are characterized by diffusive and prolonged symptoms following the initial infection. It is estimated that 10% of infected individuals experience long-term effects of COVID-19 (Ballering et al. 2022). Long-term consequences of COVID-19 present symptoms such as fatigue, cognitive impairment, cardiovascular issues, musculoskeletal complications and muti-organ damage (Ceban et al. 2022; Patel et al. 2022; Azadvari et al. 2022). A key challenge after the COVID‑19 pandemic will be to provide remediation for the affected individuals.

Current research suggests that residual viral activity and/or chronic inflammation are critical factors in the development of long COVID (Goh et al. 2022; Peluso et al. 2021). Viral infection may exacerbate age-related pathologies through enhanced SASP-mediated inflammation (Lee et al. 2021). Severe COVID-19 has indeed been associated with molecular signatures of brain aging (Mavrikaki et al. 2022). Long-COVID shares overlapping mechanistic and phenotypic properties with other post-infectious illness such as chronic fatigue syndrome, which, among other, shows premature telomere attrition indicative of accelerated cell aging (McKeever 2024; Rajeevan et al. 2018). Moreover, chronic viral infections, for instance HIV, have been linked to premature aging, a form of ‘acquired premature progeroid syndrome’ (APPS) (Grillari et al. 2007), concomitant with an early onset of age-related diseases (Breen et al. 2022; Capeau 2011 ; Filgueira et al. 2021). If age-related alteration and the accumulation of senescent cells contribute to the chronic inflammation underlying long COVID, anti-aging therapies such as senolytic and senomophic drugs, could potentially alleviate the associated symptoms and improve overall quality of life for those suffering from long COVID (Rad and Grillari 2023).

## COVID-19 and cellular senescence

Cellular senescence is a stress-induced state of permanent cell-cycle arrest that occurs in response to various stressors, including viral infections (Wang et al.^2024^). When cells enter senescence, they not only stop dividing but also begin secreting a complex mixture of pro-inflammatory cytokines, chemokines, and proteases, collectively referred to as the SASP. SARS-CoV-2, the virus responsible for COVID-19, has been shown to induce cellular senescence in host cells, a process known as virus-induced senescence (VIS) (Lee et al. 2021). This induction of senescence plays a crucial role in the pathogenesis of COVID-19 by exacerbating inflammation, causing tissue damage, and contributing to immune dysregulation. These effects are especially pronounced in elderly individuals, who typically harbor a higher burden of pre-existing senescent cells.

SARS-CoV-2 enters host cells by utilizing the ACE2 receptor. Higher levels of the ACE2 receptor were detected in primary human lung epithelial cells in vitro when exposed to the secretions from aging pre-adipocytes or endothelial cells (Camell et al. 2021). Aged hamsters exhibited higher viral load, upregulation of ACE2 in the lungs, and the colocalization of ACE2 with the senescent marker CDKN2A (p16) (Delval et al. 2023). ACE2 expression increase was observed with age in severe COVID-19 patients (Baker et al. 2021). Then, the SASP amplifies senescence effects in a paracrine way to non-ACE2 expression cells, which contributes to the pathological changes in the lungs, including excessive release of pro-inflammatory cytokines, endothelial cell damage, aberrant neutrophil activation, fibrosis, and microthrombosis, all of which are associated with severe COVID-19 outcomes (Schmitt et al. 2023). Furthermore, SARS-CoV-2-infection induce a sustained paracrine senescence phenotype and a senescence-associated inflammatory response even after the clearance of virus, suggesting that this vicious cycle might at least contribute to chronic post-COVID-19 conditions (Tsuji et al. 2022), indicating that COVID-19 might range under the senopathies (Tsuji et al. 2022; Lushchak et al. 2023)—diseases associated with senescent cells—while inflammaging and immunosenescence contribute to increased severity of COVID-19 (Zinatizadeh et al. 2022).

Aging causes the accumulation and reduced clearance of senescent macrophages, along with changes of M1/M2 macrophage ratio. In parallel, accumulation of M2 macrophages has been reported in the bronchoalveolar lavage fluid of severe COVID-19 patients, which again is associated with immune modulation and pro-fibrotic gene expression (Liao et al. 2020). Other features of the aging immune system, including decrease of T cell receptor (TCR) diversity and persistent low-grade inflammation, have also been described to occur in severe COVID-19 patients (Hou et al. 2021; Suárez-Reyes and Villegas-Valverde 2021).

**Fig. 1:**
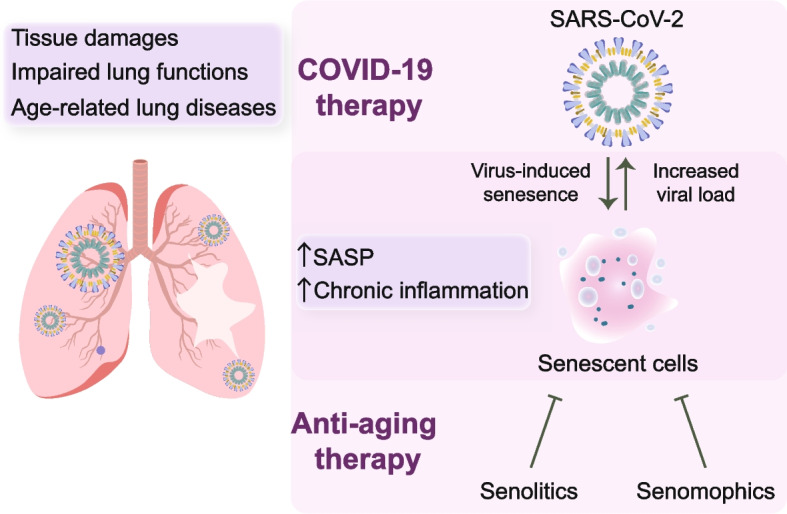
Schematic illustration describing the impact of SARS-CoV-2 on aging-associated lung dysfunction and outlining potential therapeutic strategies

Taken together, we suggest that understanding the role of senescence in COVID-19 provides valuable insights into disease mechanisms and suggests potential therapeutic opportunities. Figure 1 describes the connection between cellular senescence, anti-aging strategies and COVID-19 treatment.

## Targeting senescent cells in COVID-19 treatment

**Fig. 2:**
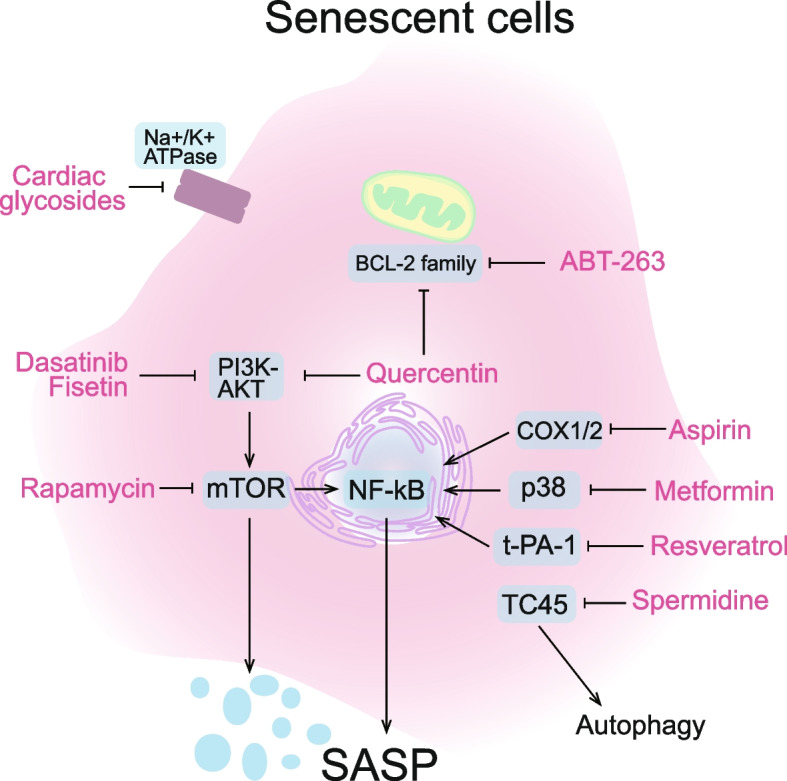
Schematic illustration of the molecular targets of senolytic and senomorphic drugs

Strategies to treat COVID-19 mainly focus on the viral replication cycle or host immune factors. Anti-aging drugs offer synergistic strategies to treat COVID-19 by targeting senescent cells, which we will discuss further in the following sections. Currently, anti-aging drugs are classified into two types: senolytics (clearance of senescent cells) – as recently reviewed by us (Rad and Grillari, 2023) and senomorphics (attenuating senescence-associated secretory phenotype) (Lagoumtzi and Chondrogianni 2021). Information of these drugs is summarized in Table 1 and the molecular targets of these drugs are represented in Fig. 2.

**Table 1:** Detailed information for senolytics and senomorphics

Drugs	Main Target	NCT#	COVID-19 and Anti-aging Evidence
Senolytics			
Dasatinib	Tyrosine kinases	NCT04115059	A phase 2 trial assess the efficacy of dasatinib in treating moderate and severe COVID-19 cases
Quercetin	PI3K	NCT04578158	A phase 2 randomized, open-labelled and controlled study aimed to investigate the adjuvant benefits of Quercetin Phytosome in community-based subjects with confirmed SARS-CoV-2 infection
Fisetin	PI3K/AKT	NCT04771611	A phase 2 placebo-controlled pilot study in COVID-19 of fisetin to alleviate dysfunction and decrease complications in at-risk outpatients
ABT-263	Bcl-2 family	Not available	Not available
Cardiac glycosides	Na + /K + -ATPase	Not available	Not available
Senomorphics			
Rapamycin	mTOR	NCT04409327	A phase 2 study to determine if RTB101 reduces the severity of COVID-19 in older adults residing in nursing homes
Metformin	NF-κB	NCT04604678	A phase 2 study into the use of metformin and LDN for patients with COVID-19
Aspirin	COX1, COX2	NCT04410328	A phase 3 study to explore the efficacy of Aggrenox (Dipyridamole ER 200 mg/ Aspirin 25 mg orally/enterally) in patients with SARS-CoV-2 infection with symptoms consistent with COVID-19
Resveratrol	t-PA-1	NCT05601180	An interventional, controlled clinical study to evaluate of the efficacy of Respicure® (resveratrol / quercetin) in the management of respiratory conditions including long COVID
Spermidine	TC45	NCT05421546	An interventional study to evaluate the efficacy of spermidine as a nutraceutical in enhancing vaccine responses, mitigating immune senescence, and reducing inflammaging among human volunteers aged over 65, either during or after vaccination against SARS-CoV-2 or influenza

### Senolytics

#### The combination of dasatinib and quercetin (D + Q)

Dasatinib, quercetin, and fisetin are classic senolytics for removal of senescent cells. These drugs can be used in combination to expand cell-type specificity and enhance their anti-aging effects. Among the most studied senolytic drugs are Dasatinib (D)—a Src/tyrosine kinase inhibitor, and Quercetin (Q)—a natural flavonoid that binds to BCL-2, when used together, they have shown promising results in vitro by effectively eliminating senescent cells (Zhu et al. 2015).

Dasatinib, a potent tyrosine kinase inhibitor (TKI), is primarily used in oncology for the treatment of chronic myeloid leukemia (CML). Recent studies have extended the application of TKIs beyond oncology and explore their potentials in treating COVID-19. Network-based analyses that integrate patient, cell line, and database information have identified TKIs like dasatinib as promising agents against COVID-19 (Tomazou et al. 2021). In silico models further support its capacity to interfere with key proteins involved in SARS-CoV-2 replication (Attia et al. 2022). Clinical observations suggest that COVID-19 patients with CML on TKI regimens not only recover faster but also exhibit a more controlled inflammatory response (Singh et al. 2021; Başcı et al. 2020; Abruzzese et al. 2020; Abdalhadi et al. 2020; Rea et al. 2020; Li 2020). Moreover, animal studies using COVID-19 models, such as K18-hACE2 mice and Roborovski dwarf hamsters, have shown encouraging outcomes with dasatinib and quercetin (D/Q) treatment (Pastor-Fernández et al. 2023), including reduced mortality rates, less pulmonary damage and fewer features of lung disease in treated viral-infected animals. This finding suggests that TKIs could have therapeutic benefits beyond their current use in cancer treatment (Lee et al. 2021).

Quercetin has been explored as a supplemental treatment for COVID-19 patients based on its antiviral properties. These properties allow Quercetin to interfere with various stages of the coronavirus entry and replication cycle, as confirmed by both in vitro and in vivo studies. Research indicates that Quercetin supplementation is safe and well-tolerated by COVID-19 patients, offering significant benefits including reduced rates of hospitalization, a decreased need for oxygen therapy, lower ICU admissions, and reduced mortality rates (Pierro et al. 2023). Moreover, Quercetin-treated patients showed quicker viral clearance and an expedited resolution of acute COVID-19 symptoms. An interventional clinical study (NCT05601180) has been conducted to evaluate the efficacy of combining resveratrol and quercetin as an add-on treatment for respiratory conditions, including long COVID in Algerian adult patients. These results suggest that Quercetin could be a valuable therapeutic agent against COVID-19, potentially on its own or in conjunction with other treatments (Agrawal et al. 2020). The expansion of D/Q into viral therapy illustrates a promising horizon for these compounds, highlighting their versatility and potential to address emergent global health challenges.

#### Fisetin

Fisetin, a flavonoid naturally found in various fruits and vegetables and available as a dietary supplement, has demonstrated potential as a senolytic agent due to its anti-inflammatory and immunomodulatory effects by targeting PI3K/AKT/mTOR signaling cascade (Sun et al. 2021; Verdoorn et al. 2021). Research involving animal models has shown that Fisetin can alleviate senescence-associated disorders in mice. In a study using a normal microbial experience mouse model infected with the β-coronavirus mouse hepatitis virus. Fisetin treatment resulted in reduced markers of senescence and decreased levels of SASP inflammatory factors in several organs, including the liver, kidney, lung, and spleen. Similar experiments using a combination of D/Q also showed significantly improved survival rates compared to control groups (Camell et al. 2021). These findings suggest that both Fisetin and D/Q can reduce senescence and inflammation post-infection, which contributes to prolonged survival and an enhanced antibody response to SARS-CoV-2.

Based on these promising results, a multicenter, placebo-controlled clinical trial has been initiated by the National Institutes of Health to further investigate Fisetin's efficacy in delaying, preventing, or treating complications associated with SARS-CoV-2 infection. This step marks a significant advancement in exploring the potential of senolytics like Fisetin in clinical settings, particularly for treating viral infections like COVID-19 (Verdoorn et al. 2021).

#### ABT-263 (navitoclax)

ABT-263, a derivative of ABT-737, was developed as a pan-BCL inhibitor and is noted for its improved oral bioavailability and aqueous solubility compared to its predecessor. Like ABT-737, ABT-263 effectively eliminates senescent cells in lung epidermis (Zhang et al. 2023). Notably, its senolytic effects have been observed to be cell-type dependent: it efficiently reduces the viability of senescent cells such as human umbilical vein epithelial cells (HUVECs), IMR90 human lung fibroblasts, and murine embryonic fibroblasts (MEFs), but it does not affect human primary preadipocytes in the same manner (Zhu et al. 2016).

In studies using aged mouse models, ABT-263 has shown promising results in reducing the susceptibility to SARS-CoV-2 infection, which is associated with the presence of age-related senescent cells. Treatment with ABT-263 has led to lower levels of SASP inflammatory factors in serum, improved lung health, and reduced markers of chronic lung disease (Delval et al. 2023). Specifically, ABT-263 treatment decreased the number of p16-positive cells, reduced senescence-associated characteristics, and lowered viral loads in aged hamsters, accompanied by decreased ACE2 expression and better lung disease outcomes. This result indicates that ABT-263 may enhance both acute and long-term outcomes of COVID-19 by clearing existing senescent cells, reducing ACE2 expression, attenuating virus replication, and alleviating lung disease features.

The effectiveness of ABT-263, however, varies across different animal models. While senolytic activity was observed in human fibroblasts, its efficacy in hamster fibroblasts infected with SARS-CoV-2 was limited (Tsuji et al. 2022). In contrast, in mouse models infected with a mouse-adapted strain of SARS-CoV-2, ABT-263 effectively decreased senescence-associated gene expression, suggesting that senolytic drugs could significantly reduce the senescence-associated inflammatory response triggered by SARS-CoV-2 infection, even after the virus has been cleared (Tsuji et al. 2022).

#### Cardiac glycosides

Cardiac glycosides (CGs), such as digoxin and ouabain, function as inhibitors of the Na + /K + ATPase and are commonly used to treat heart disease (Guerrero et al. 2019). The mechanism of CGs is rather complicated, and recent research has found their potential in treating other diseases through the modulation of autophagy. Notably, a study has found that CGs selectively kill senescent cells in mice, avoiding the toxic side effects often observed from conventional therapies (Guerrero et al. 2019). A study on the human lung adenocarcinoma cell line, A549, utilized high-throughput screening to identify CGs as potent senolytic agents, with Proscillaridin A standing out for its nanomolar range IC50 values. Similarly, Digoxin was shown to be effective against senescent cells in various cell types and under different stimuli, highlighting the broad application potentials of CGs as senolytics (Triana-Martínez et al. 2019).

Beyond their role in targeting senescent cells, CGs have also shown significant potential in combating viral infections. They inhibit the interaction between the SARS-CoV-2 spike protein and the ACE2 receptor on pulmonary and other ACE2-expressing cells, prohibiting the virus's entry into cells and reducing infection rates. Laboratory studies on drugs like ouabain, digitoxin, and digoxin support their role in disrupting crucial viral-host interactions (Caohuy et al. 2021). Further research assessing the antiviral effects of digoxin and ouabain against SARS-CoV-2 in Vero cells found that these compounds could significantly inhibit viral replication. These glycosides showed over 99% inhibition of progeny virus titers compared to control treatments, indicating effective viral suppression at the post-entry stage of the viral lifecycle (Cho et al. 2020).

The efficacy of digoxin extends beyond its antiviral activity, as it also plays a role in modulating the immune response. In studies involving cotton rats infected with an influenza strain, digoxin administration significantly reduced inflammatory factors associated with a viral-induced cytokine storm. This property could be particularly beneficial in treating severe viral infections characterized by excessive immune reaction (Pollard et al. 2020). Collectively, these findings collectively underscore the potential of Cardiac Glycosides, such as digoxin and ouabain, as multifaceted therapeutic agents capable of acting as both senolytics and antivirals, offering new avenues for the treatment of diseases like COVID-19 and beyond.

### Senomorphics

#### Rapamycin

Rapamycin is a macrolide compound known for its anti-fungal, immunosuppressive, and anti-proliferative effects wildly used in clinical research. It primarily exerts its immunosuppressive and anti-proliferative actions through inhibiting mTOR, a protein kinase that forms part of two complexes, mTORC1 and mTORC2. The mTOR-PI3K-AKT signaling pathway plays a critical role in SARS-CoV-2 infection, which can be alleviated by blocking viral genome transcription and protein synthesis (Mashayekhi-Sardoo and Hosseinjani 2022). Clinical trials are currently underway to evaluate the efficacy of drugs targeting the PI3K/Akt/mTOR pathway for COVID-19 treatment. Specifically, rapamycin targets mTORC1 more effectively, which leads to the induction of autophagy and a reversal of age-related immune-senescence through increasing the immune response (Mannick et al. 2021).

A study on age-related immune senescence in mice (22–24 months old) treated with rapamycin showed promising results. After a regimen of rapamycin or a control substance for six weeks followed by a two-week washout, the mice were immunized against H1N1 influenza. Indeed, aged mice treated with rapamycin displayed improved immune function and higher survival rates following the H1N1 challenge compared to controls, which largely failed to mount an effective immune response (Chen et al. 2009).

Moreover, it has been noted that SARS-CoV-2 infection may inhibit autophagy by disrupting various metabolic pathways. Research indicates that interventions aimed at inducing autophagy can limit the spread of SARS-CoV-2 in cell cultures. Based on these findings, rapamycin has been proposed for large-scale clinical trials as a potential treatment for COVID-19. Preliminary data indicates that low doses of rapamycin might effectively treat COVID-19, minimizing the risk of severe side effects such as cytokine storms, thus providing a potential benefit for infected patients (Husain and Byrareddy 2020).

#### Metformin

Metformin, a synthetic biguanide widely used in clinical research to treat diabetes, has emerged as a potential candidate for anti-aging intervention. Its anti-aging effects are attributed to multiple mechanisms, including suppression of cellular senescence and SASPs, inhibition of mTOR activity, and promotion of autophagy. Additionally, metformin has been observed to attenuate various age-related dysfunctions, making it a promising candidate for interventions aiming at delaying or preventing age-related diseases. The Targeting Aging with Metformin (TAME) trial, led by Dr. Nir Barzilai is currently investigating the potential of metformin to target aging and delay the onset of age-related diseases (Padki et al. 2021). This study, along with a growing body of research on metformin's anti-aging and anti-COVID-19 properties, highlights the emerging interest in repurposing this widely available and inexpensive generic drug for the purpose of addressing the challenge of multi disease.

Metformin's anti-inflammatory properties may also contribute to its potential benefits in COVID-19. In addition to inhibiting the TNFα, IL-1β and IL-6 induced Nuclear Factor kappa-light-chain-enhancer of activated B cells (NF-κB) activation, several COVID-19 drugs inhibit the NF-κB activation through disrupting the crosstalk signaling, such as MAPK (JNK/ERK/p38), ANG II-AT1R and ACE2-MAS signaling pathways (Attiq et al. 2021). For example, metformin suppresses NF-κB activation via inhibiting p38 activation and ANG II-AT1R axis (Kamyshnyi et al. 2021). This mechanism, along with its other anti-aging properties, suggests that metformin could potentially be a valuable tool in combating age-related diseases and managing COVID-19. Several clinical studies, primarily retrospective, indicating beneficial outcomes for COVID-19 patients managed with this drug (Usman et al. 2022). Insights from the TARGET-COVID study highlight metformin's association with reduced inflammation, less renal ischemia, fewer instances of thrombosis, shorter hospital stays, and lower intubation rates. Another retrospective study involving 586 patients revealed that those who used metformin prior to hospital admission experienced a 40% reduction in all-cause mortality compared to those without metformin treatment (Saygili et al. 1971). Additionally, a cohort study indicated that residents with SARS-CoV-2 infections taking metformin had a significantly lower risk of mortality within 30 days compared to those on other diabetes medications or no diabetes medications at all (Lally et al. 2021). These findings suggest that metformin's capacity to inhibit mTOR may play a role in these positive outcomes, offering a promising avenue for further research into its therapeutic benefits in COVID-19 treatment beyond traditional glycemic control.

#### Aspirin

Aspirin, commonly known as a non-steroidal anti-inflammatory drug (NSAID), has been observed to potentially extend lifespan and health span (Berkel and Cacan 2021; Strong et al. 2008). This effect may be due to its ability to delay the onset of replicative senescence in endothelial cells, likely mediated by increased nitric oxide synthesis and reduced oxidative stress, which in turn may enhance telomerase activity (Bode-Böger et al. 2005). Furthermore, the relevance of aspirin in treating COVID-19 has gained attention due to the high incidence of thromboembolic events such as venous thromboembolism and pulmonary embolism reported in COVID-19 patients, with rates as high as 42% and 17% in severe cases, respectively. Aspirin's antithrombotic properties may make it a valuable component of treatment protocols for these complications (Mohamed-Hussein et al. 2020). Aspirin's potential benefits in COVID-19 may extend beyond its antithrombotic effects. Studies have shown that aspirin inhibits NF-κB activation by inhibiting both type 1 and type 2 cyclooxygenase, leading to lower virus-induced ROS (Abani et al. 2022). This effect may contribute to aspirin's observed ability to increase the rate of being discharged alive within 28 days in COVID-19 patients, though it did not reduce mortality or the risk of progressing to invasive mechanical ventilation or death. This effect may contribute to aspirin's observed ability to increase the rate of being discharged alive within 28 days in COVID-19 patients, though it did not reduce mortality or the risk of progressing to invasive mechanical ventilation or death (Abani et al. 2022). While other antioxidant therapies, such as N-acetylcysteine, have shown promise in reducing virus-induced ROS, their clinical efficacy in severe COVID-19 patients remains unclear (Alencar et al. 2019; Hamidi et al. 2021). While other antioxidant therapies, such as N-acetylcysteine, have shown promise in reducing virus-induced ROS, their clinical efficacy in severe COVID-19 patients remains unclear (Alencar et al. 2019; Hamidi et al. 2021).

In clinical research, the impact of chronic aspirin use on COVID-19 outcomes has been investigated. A significant retrospective study involving 35,370 patients explored the effects of an active aspirin prescription prior to contracting SARS-CoV-2. The findings indicated that aspirin users had a substantially lower risk of mortality, with a 32% reduction at both 14 and 30 days post-infection (Osborne et al. 2021). Even after adjusting for variables such as age, gender, comorbidities, and mortality risk scores, aspirin use was associated with a marked decrease in mortality rates, highlighting its potential protective effect against severe outcomes of COVID-19. These insights into aspirin’s mechanisms and its potential benefits in COVID-19 treatment underscore the importance of further research to fully understand and potentially harness its therapeutic capabilities in the context of infectious diseases and age-related health challenges.

#### Resveratrol

Resveratrol, a natural compound commonly found in grapes, berries, and other plants, is known for its diverse biological activities including anti-inflammatory, anticancer, antiviral, antioxidant, cardioprotective, and neuroprotective effects. Notably, resveratrol functions as a senomorphic agent by reducing plasminogen activator-1 (t-PA-1) secretion, a molecule that facilitates fibrinolysis and activates NF-κB. This reduction in t-PA-1 indirectly modulates NF-κB activity, thereby diminishing the release of the SASP, which is implicated in the aging process and various disease states (Giordo et al. 2021).

In the context of COVID-19, a significant complication observed in severe cases is coagulopathy, which includes thrombus formation and blood vessel occlusion. Resveratrol’s anti-platelet aggregation properties could be beneficial here as well. By inhibiting platelet activation and affecting the coagulation cascade, resveratrol holds the potential to be a valuable pharmacotherapeutic agent in treating COVID-19, particularly by mitigating vascular thrombosis and systemic inflammation (Battinelli 2020; Hottz et al. 2020). Given these properties, resveratrol is considered a promising candidate for adjunct treatment in COVID-19, aiming to slow or ameliorate severe outcomes associated with the disease. This suggests a potential role for resveratrol in complementing standard COVID-19 treatments, thereby possibly enhancing patient outcomes in those experiencing severe manifestations of the virus. Further research and clinical trials could provide more definitive evidence regarding its efficacy and applications in COVID-19 treatment protocols (Giordo et al. 2021).

#### Spermidine

Spermidine has been identified to expand the life span of a series of various model organisms ranging from yeast to mouse (Eisenberg et al. 2016), a selective agonist of T-cell protein tyrosine phosphatase (TC45), plays a role in inhibiting SARS-CoV-2 replication through multiple mechanisms (Mattila et al. 2014). It promotes autophagy, which aids in degrading and removing the virus from infected cells. Additionally, spermidine may impair viral replication by inhibiting specific viral proteins. While its precise molecular mechanism is still under investigation, spermidine, has recently been studied for its potential antiviral effects against SARS-CoV-2 (Minois 2014). A recent study utilized liquid chromatography-mass spectrometry (LC–MS) to analyze amine-containing metabolites, employing a benzoyl chloride derivatization method to enhance detection sensitivity. The experimental setup for metabolic profiling and isotope tracing involved cultivating cells under various conditions, followed by incubation with either SARS-CoV-2 or a mock control. The findings from this study indicated that the exogenous administration of spermidine not only stabilized polyamine metabolism but also promoted autophagy, contributing to its antiviral effects within complex cellular systems. Specifically, spermidine treatment led to a substantial reduction in SARS-CoV-2 growth in primary human airway epithelial cells and intestinal organoids (Gassen et al. 2021). The determined half-maximal inhibitory concentration (IC50) of spermidine was 136.7 mM, suggesting that it can significantly inhibit viral growth at concentrations that are non-toxic to the cells.

These results highlight the potential of spermidine as a therapeutic agent in the treatment of COVID-19, particularly due to its dual role in modulating metabolism and enhancing the autophagic response, which together contribute to its antiviral capabilities. Further research and clinical trials could help clarify the extent of spermidine's effectiveness and its possible integration into treatment protocols for COVID-19 (Gassen et al. 2021).

## Discussion

The COVID-19 pandemic had a dramatic impact on global health and the economy. It has stimulated tremendous efforts to investigate underlying disease mechanism and to develop therapeutic interventions. Elderly individuals are more susceptible to severity outcomes of COVID-19. By investigating aging-related cellular changes and COVID-19 pathologies, scientists are exploring common mechanisms that could be targeted by anti-aging therapies. Targeted elimination of senescent cells by senolytics or suppression of SASP by senomorphics represents a promising therapeutic strategy for COVID-19.

Despite the potential of senolytic and senomorphic drugs, they are still in the early stages of clinical development, with several challenges remained to be addressed. Current senolytic and senomorphic drugs may not be universally effective across all types of senescent cells. The heterogeneity of senescent cells, which vary by tissue type and the underlying causes of senescence, necessitates the development of new compounds that can target a broader range of senescent cell types. Moreover, bioavailability issues with some senolytic and senomorphic drugs limit their broader application. While there is proof of concept for the efficacy of these therapies, translating these findings into clinical practice remains challenging. A key concern is whether anti-aging therapies directly benefit the outcome of diseases such as COVID-19, or if pleiotropic drug effects on different levels are causing the beneficial effects in COVID-19 versus on aging. Rigorous long-term and well-designed clinical trials are necessary to validate the safety and efficacy of these therapies in humans. Additionally, continued research is needed to optimize these therapies for more selective targeting of senescent cells and to explore their potential when combined with other therapeutic approaches for COVID-19 and other age-related diseases.

Interestingly, immunotherapies have also shown potential in senolytic activity. This type of therapy is widely used in cancer research and could be investigated as a senolytic approach for aging-associated diseases Novel immunological strategies, such as vaccines, CAR T-cells, and monoclonal antibodies, offer alternative approaches to senescence-targeted therapies (Du et al. 2023). While these strategies come with their own challenges and potential risks, they may help overcome certain limitations observed in current pharmacological approaches.

## Data Availability

Not applicable.

## Abbreviations

ACE2: angiotensin-converting enzyme 2
ALI: acute lung injury
AMPK: AMP kinase
ARDS: acute respiratory distress syndrome
APPS: acquired premature progeroid syndrome
CF: cystic fibrosis
CGs: Cardiac glycosides
CML: chronic myeloid leukemia
COPD: chronic obstructive pulmonary disease
COVID-19: Coronavirus disease 2019
D: Dasatinib
ECM: extracellular matrix
GLEIs: glandular-like epithelial invaginations
HUVECs: human umbilical vein epithelial cells
IC50: half-maximal inhibitory concentration
ICZ: intercartilaginous zone
IFN: interferon
JNK: JUN N-terminal kinase
LC–MS: liquid chromatography-mass spectrometry
MEFs: murine embryonic fibroblasts
MERS: Middle East respiratory syndrome
mTOR: the mammalian target of rapamycin
NSAID: non-steroidal anti-inflammatory drug
PASC: post-acute sequelae of SARS-CoV-2 infection
PCD: primary ciliary dyskinesia
Q: Quercetin
SARS: severe acute respiratory syndrome
SARS-CoV-2: syndrome coronaviruses 2
SASP: senescence associated secretory phenotype
TAME: Targeting Aging with Metformin
TKI: tyrosine kinase inhibitor
UPR: unfolded protein response
WHO: World Health Organization
